# Additional effects of erythropoietin pretreatment, ischemic preconditioning, and N-acetylcysteine posttreatment in rat kidney reperfusion injury

**DOI:** 10.3906/sag-1812-228

**Published:** 2019-08-08

**Authors:** Mohammed ELSHIEKH, Mehri KADKHODAEE, Behjat SEIFI, Mina RANJBARAN

**Affiliations:** 1 Department of Physiology, Faculty of Medicine, University of Dongola, Dongola Sudan; 2 Department of Physiology, Faculty of Medicine, Tehran University of Medical Sciences, International Campus, Tehran Iran; 3 Department of Physiology, Faculty of Medicine, Tehran University of Medical Sciences, Tehran Iran

**Keywords:** Ischemia, reperfusion, erythropoietin, ischemic preconditioning, N-acetylcysteine, oxidative stress

## Abstract

**Background/aim:**

Since the nature of ischemia/reperfusion (IR)-induced tissue damage is multifactorial and complex, in the current study, the effects of multiple treatment strategies via concomitant administration of erythropoietin (EPO) and N-acetylcysteine (NAC) with an ischemic preconditioning (IPC) regimen on renal IR injury were examined.

**Materials and methods:**

Thirty male Wistar rats were subjected to bilateral occlusion of the renal pedicles for 50 min followed by reperfusion. EPO (1000 IU/kg) was administered for 3 days, as well as IPC before the IR and NAC (150 mg/kg) administration for 4 days after IR. The animals were randomly allocated into 6 groups (n = 5): sham, IR, EPO+IR, IPC+IR, NAC+IR, and EPO+IPC+NAC+IR. Kidney tissues and blood samples were obtained for oxidative stress, proinflammatory cytokines, and renal functional evaluations.

**Results:**

IR caused significant inflammatory response, oxidative stress, and reduced renal function. Treatment with EPO, IPC, and NAC or a combination of two of them attenuated renal dysfunction and reduced the oxidative stress and inflammatory markers. Rats treated with the combination of EPO, IPC, and NAC showed a higher degree of protection compared to the other groups.

**Conclusion:**

These results showed that concomitant administration of EPO and IPC along with posttreatment NAC may have additive beneficial effects on kidney IR injury during IR-induced acute renal failure.

## 1. Introduction

Acute renal failure as a result of ischemia/reperfusion (IR) injury refers to a pathophysiological process by which renal ischemic cells become more damaged following the restoration of the blood supply [1]. IR injury as the main cause of acute renal failure occurs subsequent to different kinds of acute stress, including kidney vascular surgery, transplantation, septic shock, trauma, extracorporeal shock wave lithotripsy, or resuscitation [2]. In addition, the complex association of this pathological phenomenon with inﬂammation makes the IR injury an important risk factor for the progression of chronic kidney disease [3]. Inﬂammation, apoptosis, necrosis, and the formation of reactive oxygen species (ROS) have been suggested to be involved in the pathogenesis of IR injury [4]. The modulation of inﬂammatory response, inhibition of apoptosis, and amelioration of oxidative stress confer an advantage in the prevention and treatment of renal IR injury [5]. Several interventional mechanisms and pharmacological agents have been reported to protect renal tissues from IR injury. Since the nature of IR-induced tissue damage is complex, the protection mechanisms must be multifactorial. Thus far, a variety of mechanisms have been discussed, including free radical scavengers, reduced production of free radicals, inhibition of inflammation, and reduction of lipid peroxidation [6].

Erythropoietin (EPO) is a hematopoietic growth factor released by the kidney in response to hypoxia, inﬂammation, and cell death [7]. EPO has been suggested to exert antioxidant, antiinﬂammatory, and antiapoptotic effects against kidney IR injury [7,8].

Ischemic preconditioning (IPC), which comprises short sublethal cycles of IR prior to the prolonged ischemic insult, provides protection against renal IR injury [9]. IPC has been shown to have a strong renoprotective effect due to its antiapoptosis/necrosis, antiinflammatory, and antioxidant properties [10]. N-acetylcysteine (NAC) is able to replenish glutathione stores and scavenge oxygen free radicals. NAC has also been reported to attenuate renal IR injury by inhibiting oxidative stress [11]. However, there are no studies examining the effect of the combination of EPO and NAC with the IPC method on renal IR injury. Since the nature of IR-induced tissue damage is multifactorial and complex, the aim of present study was to investigate the effect of concomitant administration of EPO and NAC with an IPC regimen on renal IR injury. 

## 2. Materials and methods

### 2.1. Animals and diet

Male albino Wistar rats (220–270 g), from Tehran University of Medical Sciences, School of Medicine Laboratories (Iran, Tehran), were used in this study. They were maintained in an air-filtered, temperature-conditioned (25 ± 2 °C), and light-controlled (12:12 light/dark photoperiod) room. The rats were fed standard commercial pellets and water ad libitum. All of the experimental procedures were performed based on the Protection of Animals Act after approval.

### 2.2. Animal preparation

The rats were anesthetized via intraperitoneal administration of ketamine hydrochloride (50 mg/kg) and xylazine (10 mg/kg). After anesthesia, the surgical procedure for the induction of kidney IR injury was performed. Briefly, a midline abdominal incision was made, with which the abdominal cavity was fully exposed. The bilateral renal pedicles were carefully isolated and clamped using nontraumatic microvascular clamps. Throughout the induction of the kidney IR injury, the body temperature was kept between 37 and 38 °C with a heating pad. The systolic blood pressure and heart rate were continuously monitored using a tail-cuff connected to a pulse transducer device (MLT125/R; AD Instruments, Castle Hill, Australia). The transducer was connected to a PowerLab/4SP data acquisition system (Chart, Version 5; AD Instruments). After removal of the clamps, reperfusion of the kidneys was visually confirmed. The abdomen was then sutured in two layers using standard 2/0 sutures. 

### 2.3. Study design

The rats were randomly allocated into 6 groups (n = 5).

Group 1: Sham (n = 5): the rats were subjected to surgical manipulation without the induction of ischemia. 

Group 2: Ischemia/reperfusion (IR) (n = 5): the rats were subjected to bilateral renal ischemia for 50 min, followed by 3 days of reperfusion. 

Group 3: EPO (n = 5): EPO (1000 IU/kg) was administered intraperitoneally for 3 days. On day 4, the rats were subjected to 50 min of ischemia, followed by 3 days of reperfusion.

Group 4: NAC (n = 5): the rats were subjected to 50 min of ischemia, followed by 3 days of reperfusion. NAC (150 mg/kg) was administered intraperitoneally by dissolving it in normal saline for 3 days during the reperfusion period.

 Group 5: IPC (n = 5): the rats were subjected to IPC, which consisted of 3 cycles of 7 min of ischemia, followed by 7 min of reperfusion, and then subjected to 50 min of ischemia, followed by 3 days of reperfusion.

Group 6: EPO+IPC+NAC (n = 5): EPO (1000 IU/kg) was administered for 3 days. On day 4 the rats were subjected to IPC, which consisted of 3 cycles of 7 min of ischemia followed by 7 min of reperfusion, and then subjected to 50 min of ischemia followed by 3 days of reperfusion. NAC (150 mg/kg) was administered intraperitoneally from day 4 until day 7.

The rats were then placed in standard cages for the period of reperfusion. At 24 h before the end of the in vivo study, the animals were placed in metabolic cages to collect urine for urinary N-acetyl-β-(D)-glucosaminidase (NAG) activity evaluation. After 24 h, the rats were anesthetized and blood samples were obtained from the inferior vena cava. The rats were euthanized and their kidneys were harvested before being washed and dissected in cold normal saline. Part of the kidney was immediately snap-frozen in liquid nitrogen and stored at –70 °C until further use.

### 2.4. Renal function and tubular damage assessments

Plasma urea and creatinine levels were estimated using an autoanalyzer (Hitachi 704 autoanalyzer, Hitachi, Tokyo, Japan). Urine samples were collected at the end of the 24-h period and urinary NAG activity was measured on the basis of enzymatic hydrolysis of p-nitrophenyl-n-acetyl-glucosaminide using a spectrophotometer.

### 2.5. Evaluation of the renal oxidative stress

Renal oxidative stress was evaluated by measuring the activities of antioxidant enzymes superoxide dismutase (SOD) and catalase (CAT), as well as glutathione (GSH) levels, in the kidney tissues. Lipid peroxidation was evaluated by measuring the malondialdehyde (MDA) content in the kidney tissues.

### 2.6. Evaluation of the inflammatory markers

Plasma levels of tumor necrosis factor-alpha (TNF-α) and interleukin-6 (IL-6) were evaluated using a commercial enzyme-linked immunosorbent assay (ELISA) kit (R&D Systems Inc., USA).

### 2.7. Statistical analysis

SPSS 20.0 (IBM Corp., Armonk, NY, USA) was used for data analysis. Differences among the groups were assessed with one-way ANOVA and the Tukey HSD test was used to examine the difference between two groups. Statistical significance was accepted at P < 0.05; the arithmetic means ± standard errors of the means were used to define the distribution. 

## 3. Results

### 3.1. EPO, IPC, NAC, and their combination attenuated postischemic renal dysfunction

**Figure 1:**
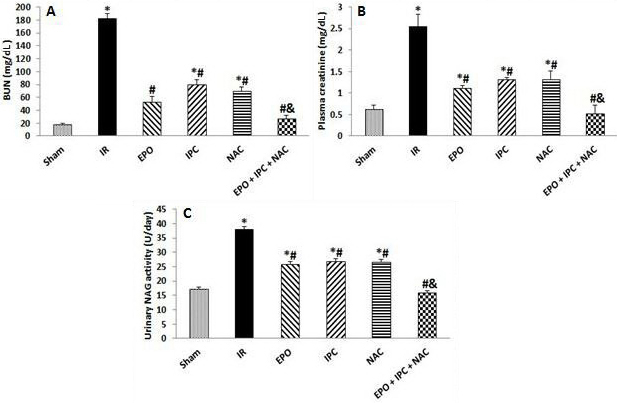
Plasma BUN (A) and creatinine (B) levels, and urinary NAG (C) activity. Results are presented as the mean ± standard error of the mean (SEM); n = 5 in each group: *P < 0.05 vs. Sham group; #P ˂ 0.05 vs. IR group; &P < 0.05 vs. EPO, IPC, and NAC groups.

As demonstrated in Figures 1A and 1B, the renal functional markers (BUN and Cr) of the animals subjected to IR showed significant increases compared to the Sham group (P < 0.05). These markers were significantly improved by treatment with EPO, IPC, and NAC when compared to the IR group (P < 0.05). However, in the combination group, the functional parameters were significantly lower than in the other treated groups (P < 0.05), suggesting that a combination of EPO, IPC, and NAC was more effective on IR-induced renal dysfunction.

### 3.2. EPO, IPC, NAC, and their combination reduced postischemic tubular damage

In the IR group, urinary NAG activity was significantly elevated when compared to the Sham group (P < 0.05). Treatment with EPO, IPC, and NAC alone significantly reduced tubular damage when compared to the IR group (P < 0.05). However, in the combination group, the tubular damage was significantly lower than in the other treated groups (P < 0.05), suggesting that a combination of EPO, IPC, and NAC was more effective on IR-induced renal tubular injury (P < 0.05, Figure 1C).

### 3.3. EPO, IPC, NAC, and their combination attenuated postischemic oxidative stress

**Figure 2:**
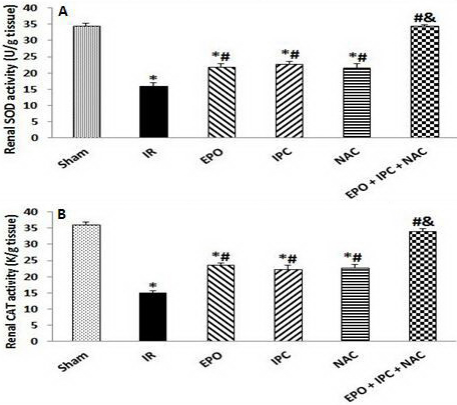
Renal tissue SOD (A) and CAT (B) activities. Results are presented as the mean ± standard error of the mean (SEM); n = 5 in each group: *P < 0.05 vs. Sham group; #P < 0.05 vs. IR group; &P < 0.05 vs. EPO, IPC, and NAC groups.

Renal IR injury significantly decreased the renal tissue SOD and CAT activities when compared to the Sham group (P < 0.05). Treatment with EPO, IPC, and NAC alone resulted in significantly higher activities of SOD and CAT when compared to the IR group (P < 0.05). However, the activities of these enzymes were significantly higher in the combination group than in the other treated groups (P < 0.05), suggesting that a combination of EPO, IPC, and NAC was more effective on IR-induced renal oxidative stress (P < 0.05, Figures 2A and 2B). 

**Figure 3:**
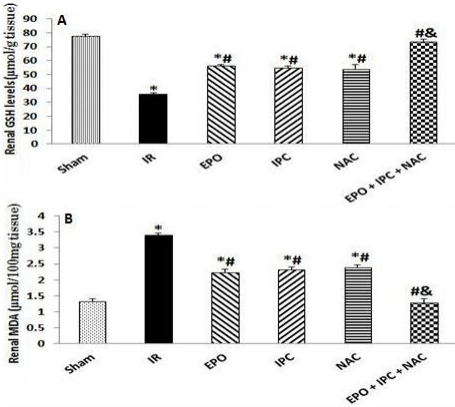
Renal tissue GSH levels (A) and MDA contents (B). Results are presented as the mean ± standard error of the mean (SEM); n = 5 in each group: *P < 0.05 vs. Sham group; #P < 0.05 vs. IR group; &P < 0.05 vs. EPO, IPC, and NAC groups.

The renal tissue GSH level was significantly reduced in the IR group when compared to the Sham group (P < 0.05). Treatment with EPO, IPC, and NAC alone resulted in a significantly higher GSH level when compared to the IR group (P < 0.05). However, the GSH concentration was significantly higher in the combination group than in the other treated groups (P < 0.05), suggesting that a combination of EPO, IPC, and NAC was more effective on IR-induced renal oxidative stress (P < 0.05, Figure 3A).

Renal IR injury resulted in a significant elevation in the renal tissue MDA content when compared to the Sham group (P < 0.05). Treatment with EPO, IPC, and NAC alone resulted in a significantly lower MDA level when compared to the IR group (P < 0.05). However, the MDA concentration was significantly lower in the combination group than in the other treated groups (P < 0.05), suggesting that a combination of EPO, IPC, and NAC was more effective on IR-induced renal oxidative stress (P < 0.05, Figure 3B).

### 3.4. EPO, IPC, NAC, and their combination suppressed postischemic inflammatory cytokines

**Figure 4:**
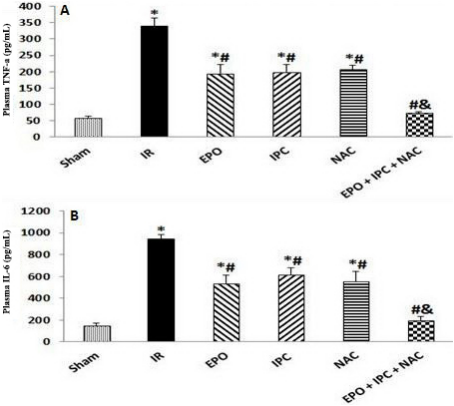
Plasma TNF-α (A) and IL-6 (B) levels. Results are presented as the mean ± standard error of the mean (SEM); n = 5 in each group: *P < 0.05 vs. Sham group; #P < 0.05 vs. IR group; &P < 0.05 vs. EPO, IPC, and NAC groups.

Renal IR injury resulted in significant elevations in plasma TNF-α and IL-6 when compared to the Sham group (P < 0.05). Treatment with EPO, IPC, and NAC alone resulted in significantly lower plasma TNF-α and IL-6 when compared to the IR group (P < 0.05). However, these parameters were significantly lower in the combination group than in the other treated groups (P < 0.05), suggesting that the combination of EPO, IPC, and NAC was more effective on IR-induced renal (P < 0.05, Figures 4A and 4B).

## 4. Discussion 

Due to the multifactorial nature of IR-induced kidney damage, this study was designed to investigate the effect of concomitant administration of EPO and NAC with an IPC regimen on renal IR injury. The findings showed that pretreatment with EPO, IPC insults, and posttreatment with NAC ameliorated the organ damage in a bilateral renal IR injury rat model, with the greatest effects seen in the combination group. The kidney is extremely sensitive to hypoxia, which makes it vulnerable to ischemic injury. Oxidative stress is considered as the key step in the initiation and development of renal IR injury [12]. Severe oxidative stress can make renal transplantation grafts highly prone to acute and chronic rejection [13]. Under these conditions, ROS are produced by the mitochondrial respiratory chain in the ischemia phase and magnified in the reperfusion period, which may eventually result in cell death by directly impairing DNA, proteins, and lipids [14]. IR injury induces the release of the proinflammatory cytokines and chemokines from the kidney vasculature and parenchymal cells [15]. In the present study, renal functional impairments were evidenced by significantly increased plasma urea, creatinine, inflammatory cytokines, urinary NAG activity, and oxidative stress markers, as well as a significant reduction in renal tissue antioxidant enzyme activities in the bilateral renal IR group.

EPO is a kidney-derived growth factor that has long been considered as the stimulating factor for erythropoiesis. However, recent studies have reported that EPO may exert protective effects against IR injury, by the attenuation of renal dysfunction, oxidative stress, inﬂammatory response, and cell death [16,17]. Our results showed that pretreatment with EPO alone or in combination with IPC and posttreatment NAC reduced the renal tissue MDA content and elevated renal tissue SOD and CAT activities and GSH levels, and then subsequently reduced kidney dysfunction (plasma urea, Cr, and urinary NAG activity). These results were consistent with our previous studies, which demonstrated the antioxidant effects of EPO on renal IR injury [16]. The antioxidant effect of EPO may be related to its ability to induce the formation of antioxidant enzymes, such as hemoxygenase-1 [18] and glutathione peroxidase [19].

In the current study, pretreatment with EPO alone or in combination with IPC and NAC significantly suppressed the plasma levels of proinflammatory cytokines TNF-α and IL-6. This was in agreement with previous studies, which demonstrated that EPO treatment suppressed the inflammatory response in IR injury by reducing proinflammatory cytokines, such as TNF-α and IL-6 [16,20]. 

IPC has been shown to have an ameliorative effect on kidney IR injury due to its antiapoptotic, antiinflammatory, and antioxidant properties [9,10]. In this study, it was found that application of IPC alone or in combination with EPO and NAC significantly attenuated kidney damage. In agreement with these findings, several studies have demonstrated the renoprotective effects of IPC on kidney IR injury [16,21].

TNF-α, in association with IL-17A, induces the expression of transcriptional IL-6 and other proinﬂammatory cytokines and participates in an inﬂammatory response to kidney IR injury [22,23]. TNF-α, which is expressed in renal tubular cells, participates in the infiltration of various immune cells to create a positive feedback cycle of inflammatory response [22]. In the present study, plasma TNF-α and IL-6 levels were significantly suppressed in the treatment groups when compared to the IR group, with the greatest effect seen in the combination group. Consistent with these results, IPC exhibited a potent antiinﬂammatory capacity to suppress kidney IR injury by reducing the plasma levels of TNF-α and IL-6 [24].

In this study, the possible protective effect of NAC alone or in combination with EPO and IPC was evaluated. The results demonstrated that posttreatment with NAC alone or in combination with EPO and IPC exhibited an attenuation effect on kidney IR injury. NAC attenuates oxidative stress in renal IR injury by the direct scavenging of ROS, such as hypochlorous acid, hydrogen peroxide, superoxide, and the hydroxyl radical [25], and by the prevention of lipid peroxidation and protection of GSH contents [26]. In a recent study, NAC was demonstrated to have ameliorative effects on renal IR injury [27]. Plasma levels of TNF-α and IL-6 have been widely used as indices for proinflammatory response. In the present study, posttreatment with NAC alone or in combination with a pretreatment of EPO and IPC significantly reduced the plasma levels of TNF-α and IL-6 when compared to the IR group, with the greatest effect seen in the combination group. NAC is a free radical scavenger, which in acute renal injury has ameliorative effects on inflammatory responses, such as the inhibition of cytokine expression and the suppression of NF-κB [28]. In agreement with this study, the inhibition of proinflammatory cytokines by NAC was demonstrated in an animal model of acute kidney injury [27].

In conclusion, these results showed that concomitant administration of EPO and IPC along with NAC posttreatment may have an additive beneficial effect on kidney IR injury during IR-induced acute renal failure.

## References

[ref0] (2014). Expression of Bcl-2 and NF-κB in brain tissue after acute renal ischemia-reperfusion in rats. Asian Pacific Journal of Tropical Medicine.

[ref1] (2011). Autophagy protects the proximal tubule from degeneration and acute ischemic injury. Journal of the American Society of Nephrology.

[ref2] (2011). Cellular pathophysiology of ischemic acute kidney injury. Journal of Clinical Investigation.

[ref3] (2006). Update on mechanisms of ischemic acute kidney injury. Journal of the American Society of Nephrology.

[ref4] (2015). Acute kidney injury: preclinical innovations, challenges, and opportunities for translation. Canadian Journal of Kidney Health and Disease.

[ref5] (2016). The effects of tadalafil and pentoxifylline on apoptosis and nitric oxide synthase in liver ischemia/reperfusion injury. Kaohsiung Journal of Medical Sciences.

[ref6] (2018). Renoprotective effect of erythropoietin via modulation of the STAT6/MAPK/NF-κB pathway in ischemia/reperfusion injury after renal transplantation. International Journal of Molecular Medicine.

[ref7] (2015). Protection against ischemia/reperfusion induced renal injury by cotreatment with erythropoietin and sodium selenite. Molecular Medicine Reports.

[ref8] (2015). Remote ischemic preconditioning for kidney protection: GSK3β-centric insights into the mechanism of action. American Journal of Kidney Diseases.

[ref9] (2012). Modulation of apoptosis by ischemic preconditioning: an emerging role for miR-21.

[ref10] (2006). N-acetylcysteine attenuates kidney injury in rats subjected to renal ischaemia-reperfusion. Nephrology Dialysis Transplantation.

[ref11] (2016). Molecular dissection of renal ischemia-reperfusion: oxidative stress and cellular events. Current Medicinal Chemistry.

[ref12] (2011). Oxidative stress in kidney transplantation: causes, consequences, and potential treatment. Iranian Journal of Kidney Diseases.

[ref13] (2014). Mitochondrial reactive oxygen species: a double edged sword in ischemia/reperfusion vs. preconditioning. Redox Biology.

[ref14] (2004). Ischemic acute renal failure: an inflammatory disease. Kidney International.

[ref15] (2017). Up-regulation of nitric oxide synthases by erythropoietin alone or in conjunction with ischemic preconditioning in ischemia reperfusion injury of rat kidneys. General Physiology and Biophysics.

[ref16] (2013). Protective effects of erythropoietin on endotoxin-related organ injury in rats. Journal of Huazhong University of Science and Technology-Medical Sciences.

[ref17] (2007). Erythropoietin induces heme oxygenase-1 expression and attenuates oxidative stress. Biochemical and Biophysical Research Communications.

[ref18] (2007). The relationship between erythropoietin pretreatment with blood-brain barrier and lipid peroxidation after ischemia/reperfusion in rats. Life Sciences.

[ref19] (2005). Protective effect of erythropoietin on renal ischemia and reperfusion injury. ANZ Journal of Surgery.

[ref20] (2014). Activation of nuclear factor erythroid 2-related factor 2 (Nrf2) and Nrf-2-dependent genes by ischaemic pre-conditioning and post-conditioning: new adaptive endogenous protective responses against renal ischaemia/reperfusion injury. Acta Physiologica.

[ref21] (2002). IL-6 secretion by human pancreatic periacinar myofibroblasts in response to inflammatory mediators. Journal of Immunology.

[ref22] (2015). Activation of Nrf2 by ischemic preconditioning and sulforaphane in renal ischemia/reperfusion injury: a comparative experimental study. Physiological Research.

[ref23] (2001). Differential cellular immunolocalization of renal tumour necrosis factor-alpha production during ischaemia versus endotoxaemia. Immunology.

[ref24] (2008). The influence of N-acetyl-L-cysteine on oxidative stress and nitric oxide synthesis in stimulated macrophages treated with a mustard gas analogue. BMC Cell Biology.

[ref25] (2009). Protective effect of sulfhydryl-containing antioxidants against ischemia/reperfusion injury of prepubertal rat intestine. Journal of Gastroenterology and Hepatology.

[ref26] (2014). Effects of dexpanthenol and N-acetylcysteine pretreatment in rats before renal ischemia/reperfusion injury. Renal Failure.

